# The novel biomarkers for assessing clinical benefits of continuous renal replacement therapy in pediatric sepsis: a pilot study

**DOI:** 10.1186/s12014-023-09392-2

**Published:** 2023-01-18

**Authors:** Yun Cui, Shuyun Feng, Huijie Miao, Tiantian Liu, Jingyi Shi, Jiaying Dou, Chunxia Wang, Yucai Zhang

**Affiliations:** 1 Department of Critical Care Medicine, Shanghai Children’s Hospital, Shanghai Jiao Tong University School of Medicine, Shanghai, 200062 China; 2 Institute of Pediatric Infection, Immunity, and Critical Care Medicine, Shanghai Children’s Hospital, Shanghai Jiao Tong University School of Medicine, Shanghai, 200062 China; 3 Institute of Pediatric Critical Care, Shanghai Jiao Tong University, Shanghai, 200062 China; 4 Clinical Research Unit, Shanghai Children’s Hospital, Shanghai, 200062 China

**Keywords:** Continuous renal replacement therapy, Pediatric sepsis, Biomarker, Serum proteomics

## Abstract

**Background:**

Continuous renal replacement therapy (CRRT) has been considered as an adjuvant therapy for sepsis. However, the novel biomarker to evaluate the benefits of CRRT is limited. The aim of this study was to explore the novel biomarkers involved in the impact of CRRT in pediatric sepsis.

**Methods:**

The serum proteomic profiles on the 7th day after CRRT (CRRT 7th day) compared with before CRRT (CRRT 1st day) was determined in 3 children with sepsis as a discovery set. The screened candidates were confirmed in the validation cohort including patients received CRRT (CRRT group) and without CRRT (non-CRRT group). We defined that pediatric sequential organ failure assessment score (pSOFA) in pediatric patients with sepsis decreased by 2 points or more on the CRRT 1st day compared with CRRT initiation as CRRT responders. The changes of serum biomarkers were compared between CRRT responders and CRRT non-responders. Moreover, correlation analysis was further conducted in pediatric sepsis.

**Results:**

A total of 145 differentially expressed proteins were found according to the serum proteomics profiles. By visualizing the interaction between the differential proteins, 6 candidates (Lysozyme C [LYZ], Leucine-rich alpha-2-glycoprotein [LRG1], Fibromodulin [FMOD], Alpha-1-antichymotrypsin [SERPINA3], L-selectin [SELL], Monocyte differentiation antigen CD14 [CD14]) were screened. In the validation cohort, serum levels of LYZ and LRG1 showed a higher trend on the CRRT 7th day than that on the 1st day in the non-CRRT group. However, the changes in levels of LYZ and LRG1 on the 7th day was significant in the CRRT group (p = 0.016, p = 0.009, respectively). Moreover, the levels of LYZ and LRG1 on the CRRT 7th day in the CRRT group were significantly higher than that in the non-CRRT group (p < 0.001, p = 0.025). Decreased levels of CD14 were associated with sepsis recovery, but not associated with CRRT. There were no significantly difference in serum FMOD, SERPINA3, and SELL levels. Importantly, serum LYZ and LRG1 levels changed in CRRT responders, but not CRRT non-responders. Further analysis indicated that serum LYZ levels were correlated to total platelet counts, aspartate aminotransferase (ALT), alanine aminotransferase (AST), and albumin levels, and serum LRG1 level were correlated to total platelet count and TBIL levels on the 1st day in the CRRT group. Protein–protein interaction network analysis displayed that serum LYZ and LRG1 were involved in the process of inflammatory response, leucocytes adhesion to vascular endothelial cell, as well as complement activation.

**Conclusion:**

Elevated serum LYZ and LRG1 levels are associated with clinical benefits of CRRT during sepsis.

**Supplementary Information:**

The online version contains supplementary material available at 10.1186/s12014-023-09392-2.

## Introduction

Sepsis is a life-threatening organ dysfunction caused by a dysregulated host response to infection [1]. Previous studies reported that about 10–40% patients suffered from sepsis admitted to intensive care unit (ICU) [2–4], and about 25–30% develop sepsis within 24 h in ICU [5–7]. The hospital mortality rates for sepsis range from 15 to 35% in the ICU [3]. Therefore, early recognition and management are essential to optimize the prognosis of children with sepsis. Levels of cytokines (such as interleukin-8 [IL-8], tumor necrosis factor -α [TNF-α], IL-10, and IL-6) and endotoxins are often elevated in patients with sepsis [8, 9].

Continuous renal replacement therapy (CRRT) is regarded as an adjuvant therapy for critically ill, which removes excess fluids and clear uremic toxins, endotoxins, proteins, and inflammatory mediators efficiently [10]. In patients with sepsis, CRRT has the advantages of maintaining fluid balance, steady acid–base, and electrolyte correction [11]. “Surviving Sepsis Campaign (SSC) international guidelines” for children in 2020 suggested that CRRT was considered in hemodynamically unstable septic patients for promoting management of fluid balance and acute kidney injury (AKI) [12]. CRRT removing endotoxins and cytokines from septic patients is associated with improved patient outcomes [13]. Our previous multicenter study indicated that the level of IL-6 was decreased in the CRRT group compared with the non-CRRT group, and CRRT decreases hospital mortality rate in pediatric severe sepsis, especially in patients with acute respiratory distress syndrome (ARDS) [14]. Consistently, a recent study showed that levels of C-reactive protein (CRP), TNF-α, and IL-6 decreased significantly after CRRT in patients with sepsis-induced acute kidney injury (AKI) [15]. CRRT as an extracorporeal life support could disturb homeostasis and affect the process of sepsis. However, there is limited reports about the integrated information using proteomic profiling technology to reveal the effects of CRRT in sepsis.

In this study, the changes of serum proteomics profiles before and after CRRT for 7 days were analyzed in 3 pediatric sepsis as a discovery set. After integrating the information based on ingenuity pathway analysis (IPA) and protein–protein interaction (PPI) network analysis,6 candidates were screened and further validated by enzyme linked immunosorbent assay (ELISA) in a larger-scale independent sample set. The aim of this study was to explore the valuable biomarkers for assessing the clinical benefits of CRRT in pediatric sepsis.

## Materials and methods

### Study design and patients’ enrollment

Patients with severe sepsis were enrolled in pediatric ICU at Shanghai Children’s Hospital from January 2020 to December 2021. The inclusion criteria were as follows: (1) aged over 28 days to 18 years old; (2) diagnosed with severe sepsis according to the International Pediatric Sepsis Consensus Conference in 2005 [16]. The exclusion criteria included (1) patients with genetic disease, heredity metabolic disease; (2) length of PICU stay less than 7 days; (3) patients with advanced tumor. For patients who had repeated admissions to PICU, only the first PICU admission was included for further analysis. The research protocol conformed to the ethical guidelines of the 1975 Declaration of Helsinki was approved by the Ethics Committee of the Children’s Hospital affiliated to Shanghai Jiao Tong University (2018R039-E02, 2018R039-E03, 2018R039-E04). The informed consent to both CRRT support during PICU hospitalization and enrollment into this study was provided by the participants’ parents or relatives.

### Clinical improvement

A pediatric version of the Sequential Organ Failure Assessment score (pSOFA) was used to assess the clinical improvement of patients in the validation set [17]. We defined that pSOFA score in pediatric patients with sepsis decreased by 2 points or more on the CRRT 1st day (CRRT 1st day) compared with CRRT initiation (pre-CRRT) as CRRT responders.

### Blood sampling

In the discovery set, serum was collected in patients with severe sepsis before CRRT (CRRT 1st day) and after CRRT for 7 days (CRRT 7th day) in sterile vacutainer tubes for further analysis. In the validation set, serum was collected in patients with severe sepsis at the time of admission to PICU as 1st day and on the 7th day after admission as the 7th day in the non-CRRT group. In the CRRT group, serum was collected before CRRT as 1st day and on the 7th day after CRRT as 7th day. In the CRRT group, serum was collected before CRRT as 1st day and on the 7th day after CRRT as 7th day. To avoid the bias due to course of sepsis or therapy, the interval time between CRRT initiation and PICU admission was set within 24 h in this study. Usually, serum from the residual blood of healthy children for routine patient care were stored at − 80 °C after the results of clinical detection were confirmed. Moreover, the personal information about these residual blood samples were anonymized. Serum was obtained and stored at – 80 ℃ for further analysis.

### Data collection

Medical records were reviewed to extract the demographic data (such as age, gender, weight), details of clinical features, and Pediatric Risk of Mortality score III (PRISM III on PICU admission. Laboratory parameters including white blood cells (WBC), procalcitonin (PCT), CRP, total bilirubin(TBIL), alanine aminotransferase (ALT), γ-Gglutamyltransferase (γ-GT), creatinine (Cr), and blood urea nitrogen (BUN), serum lactate (Lac), albumin (Alb) and coagulation indicators such as international normalized ratio (INR), fibrinogen (Fib), anginal partial thromboplastin time (APTT), prothrombin time (PT) and platelet (PLT) were obtained on the 1st day and 7th day as same as blood sampling both in the CRRT group and the non-CRRT group.

### Proteomics analysis and GO enrichment for differential expressed proteins

The Multiple Affinity Removal LC Column-Human 14 (Cat. 5188-6560, Agilent, CA, USA) was used to remove the high-abundance protein in the serum according to the corresponding protocol of the manufacturer to obtain a low-abundance component solution. Samples were lysed with SDT buffer (4% SDS, 100 mM DTT, 150 mM Tris–HCl pH 8.0) to extract proteins. Used the BCA Protein Assay Kit (Bio-Rad, Hercules, CA, USA) to quantify the protein. An appropriate amount of protein from each sample was digested by trypsin using the Filter aided proteome preparation (FASP). A total of 100 μg peptide mixture of each sample was labeled using TMT reagent according to the manufacturer’s instructions (Thermo Fisher Scientific, Waltham, MA, USA). The labeled peptides of each group were mixed in equal amounts and fractionated using the High pH Reversed-Phase Peptide Fractionation Kit. LC–MS/MS analysis was performed on a Q Exactive mass spectrometer (Thermo Scientific) that was coupled to Easy nLC (Proxeon Biosystems, now Thermo Fisher Scientific). The mass spectrometry proteomics data have been deposited to the ProteomeXchange Consortium (http://proteomecentral.proteomexchange.org) via the iProX partner repository with the dataset identifier PXD032807 [18].

Blast2GO (version 3.3.5) was searched during the gene ontology mapping and annotation procedure [19]. GO enrichment was applied based on a *Fisher’* exact test, with *p* < 0.05 considered as statistically significant functional categories. Compared with the sepsis group, final proteins considered to be differentially expressedand chosen for further analysis by *p* < 0.05 and 1.2-foldchanges (> 1.20 or < 0.83) for proteomic analysis [20].

### Ingenuity pathway analysis and protein–protein interaction (PPI) network analysis

The analyses of canonical pathways, bio-functions and the networks were conducted by using the IPA system (IPA, http://www.ingenuity.com) for the candidate proteins to gain further insights into the typical alterations. Furthermore, molecular interactions between differentially expressed proteins were annotated by STRING database (https://string-db.org/) and visualized by Cytoscape v3.9.1.

### Serum candidates analysis using enzyme-linked immunosorbent assay

The concentration of serum monocyte differentiation antigen CD14 (CD14), leucine-rich alpha-2-glycoprotein (LRG1), fibromodulin (FMOD), L-selectin (SELL), lysozyme C (LYZ), and alpha-1-antichymotrypsin (SERPINA3) was determined using ELISA kit (Multi Sciences Co. Ltd, Cat No. 70-EK1101-96, 70-EK185-96, 70-EK1290-96, 70-EK185-96, 70-EK1101-96. Shanghai Lengton Bioscience Co. Ltd BPE10440, BPE11483, BPE10339, BPE12434, BPE10437, BPE11291).

### Statistical analysis

The continuous data with normal distribution were presented as mean ± standard deviation (SD); otherwise, they were expressed as median (interquartile range, IQR). The comparison between two groups were conducted using the Student’s *t* test for the continuous data with normal distribution or Mann–Whitney *U*-test for data with abnormal distribution, respectively. *Pearson* correlation analysis was performed to analyze the correlation between novel biomarkers and clinical or laboratory index. All statistical tests were two-tailed and a value of *p* < 0.05 was considered statistically significant. All statistical analyses were performed using SPSS 22.0 software (IBM, Armonk, NY, USA).

## Results

### Baseline characteristics of patients in the validation set

**Fig. 1:**
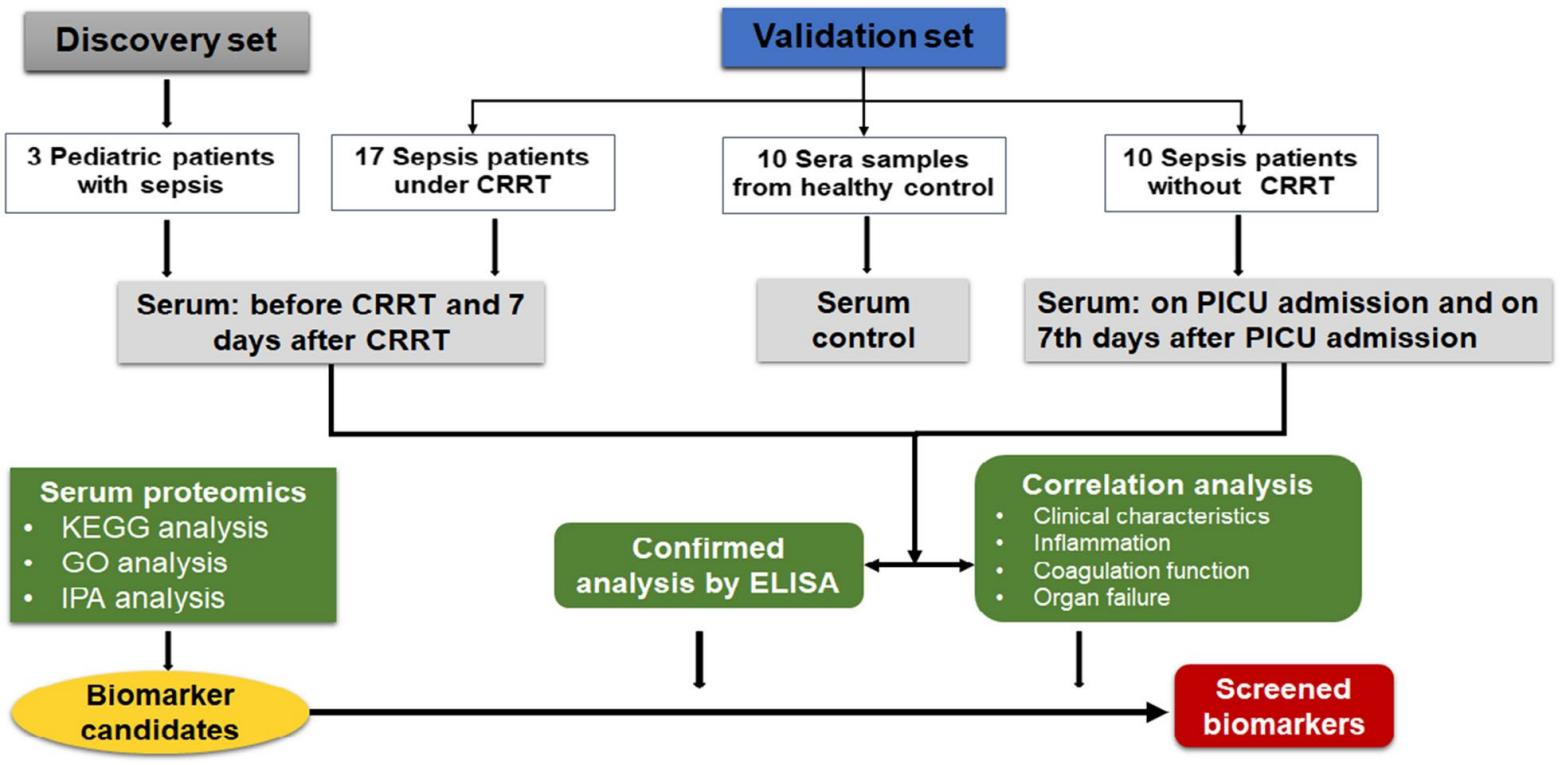
Flowchart of experimental design for biomarker screening

A total of 27 sepsis patients received CRRT and 19 sepsis patients without CRRT were eligible. To match the severity of patients in the non-CRRT group, 8 patients dead within 7 days after PICU admission were excluded, and 2 patients included in the discovery set were also excluded. Finally, 17 cases were entered into the validation set as the CRRT group. In the non-CRRT group, patients with advanced tumor (n = 3), congenital heart disease (n = 3), or the concentration of biomarkers exceeded the ELISA KIT detection range(n = 3) were excluded. Eventually, 10 sepsis patients without CRRT were included in the validation set as the non-CRRT group. Sera from residual blood after physical examination from 10 healthy children were used as control. In the present study, subjects were divided into discovery set including 3 pediatric patients with severe sepsis received CRRT, and the validation set including 10 healthy controls, 10 patients with severe sepsis without CRRT, and 17 patients with severe sepsis under CRRT, which was schematically summarized in Fig. 1.

The median (IQR) age of pediatric patients admitted to the PICU in this cohort was 65 (33.5, 113.5) months in the CRRT group and 44.5 (4.75, 117.25) months in the non-CRRT group. In the validation set, 47% in the CRRT group and 50% in the non-CRRT group were male. The values of APTT were 80.70 (38.6, 155.6) s in the CRRT group and36.1(28.98,38.25) s in the non-CRRT group, respectively (*p* = 0.015). In addition, there were no significant differences in aspect of weight, age, gender, PRISM III, CRP, WBC, PCT, ALT, TBIL, etc*.* (Table 1).

**Table 1:** Baseline characteristics of patients in the validation group

Characteristics	Total (n = 27)	CRRT (n = 17)	non-CRRT (n = 10)	*P* value
Body weight, kg	17.75 (12.38, 32.40)	19 (15, 28)	15 (8.58, 45)	0.372
Age, month	64 (24, 114)	65 (33.5, 113.5)	44.5 (4.75, 117.25)	0.436
Male, n (%)	13 (48%)	8 (47%)	5 (50%)	> 0.999
PRISM III	2.50 (0.75, 4.50)	1.5 (0.25, 4)	4 (1.5, 6)	0.296
CRP, mg/L	100 (54, 160)	89 (45.5,154)	137 (65.75, 160)	0.420
WBC, × 10^9^ /L	8.25 (3.99,13.70)	8.25 (4.64,14.07)	7.58 (3.03,14.45)	0.673
PCT, ng/mL	1.06 (0.22, 12.67)	0.52 (0.2, 2.54)	10.48 (0.51, 42.93)	0.088
ALT, U/L	34 (25, 57.50)	51 (24, 60)	30.5 (26, 74.75)	0.739
TBIL, μmol/L	20.31 (10.13,27.41)	25.08 (9.65,31.95)	12.80 (9.78,24.48)	0.108
γ-GT, U/L	20.50 (11.25, 96.5)	17 (11, 82)	63 (15.5, 135)	0.200
Fib, g/L	2.97 (2.06, 4.67)	2.73 (1.89, 4.19)	3.81 (2.74, 7.10)	0.092
APTT, s	39.85 (35.53, 143.15)	80.7 (38.6, 155.6)	36.1 (28.98, 38.25)	0.015^a^
INR	1.39 (1.21, 1.55)	1.37 (1.20, 1.76)	1.40 (1.26, 147)	0.916
PT, s	15 (13.53, 17.10)	15.35 (13.75, 19.05)	14.8 (11.95, 16.15)	0.444
PLT, × 10^9^ /L	161 (77,274)	161 (83.5,255.5)	171.5 (43.5,327.5)	0.725
Cr, μmol/L	29 (25.5, 52.6)	29 (25, 35)	27.8 (25.28, 62.43)	0.598
BUN, mmol/L	4.10 (2.75,8.90)	3.90 (2.10,5.20)	7.07 (3.75,11.00)	0.063
Alb, g/L	32.31 (29.70,38.25)	31.58(26.60,35.62)	34.30(31.25,40.175)	0.120
Lac, mmol/L	2.15 (1.10,2.80)	2.5 (1.45, 3.05)	0.9 (0.7, 2.2)	0.061

*PRISM III* pediatric risk of mortality III, *CRP* c-reactive protein, *WBC* white blood cell, *PCT* procalcitonin, *ALT* alanine aminotransferase, *TBIL* bilirubin, *γ-GT* γ-glutamyl transpeptidase, *Fib* fibrinogen, *APTT* activated partial thromboplastin time, *INR* international normalized ratio, *PT* prothrombin time, *PLT* platelet, *Cr* creatinine, *BUN* blood urea nitrogen, *Alb* albumin, *Lac* lactate

^a^*P* < 0.05

### Clinical improvement of patients in the validation set

To assess the clinical improvement of patients in CRRT group and non-CRRT group, the pSOFA score were determined at the indicated time. The changes of pSOFA of patients either in CRRT or non-CRRT group have a significantly decrease on 7th day compared with pre-CRRT or on PICU admission, respectively (Additional file 1: Table S1). Moreover, there were 10 CRRT responders and 7 CRRT non-responders according to the definition of validity for CRRT in this study (Additional file 1: Table S2).

### TMT-based proteomic analyses

**Fig. 2:**
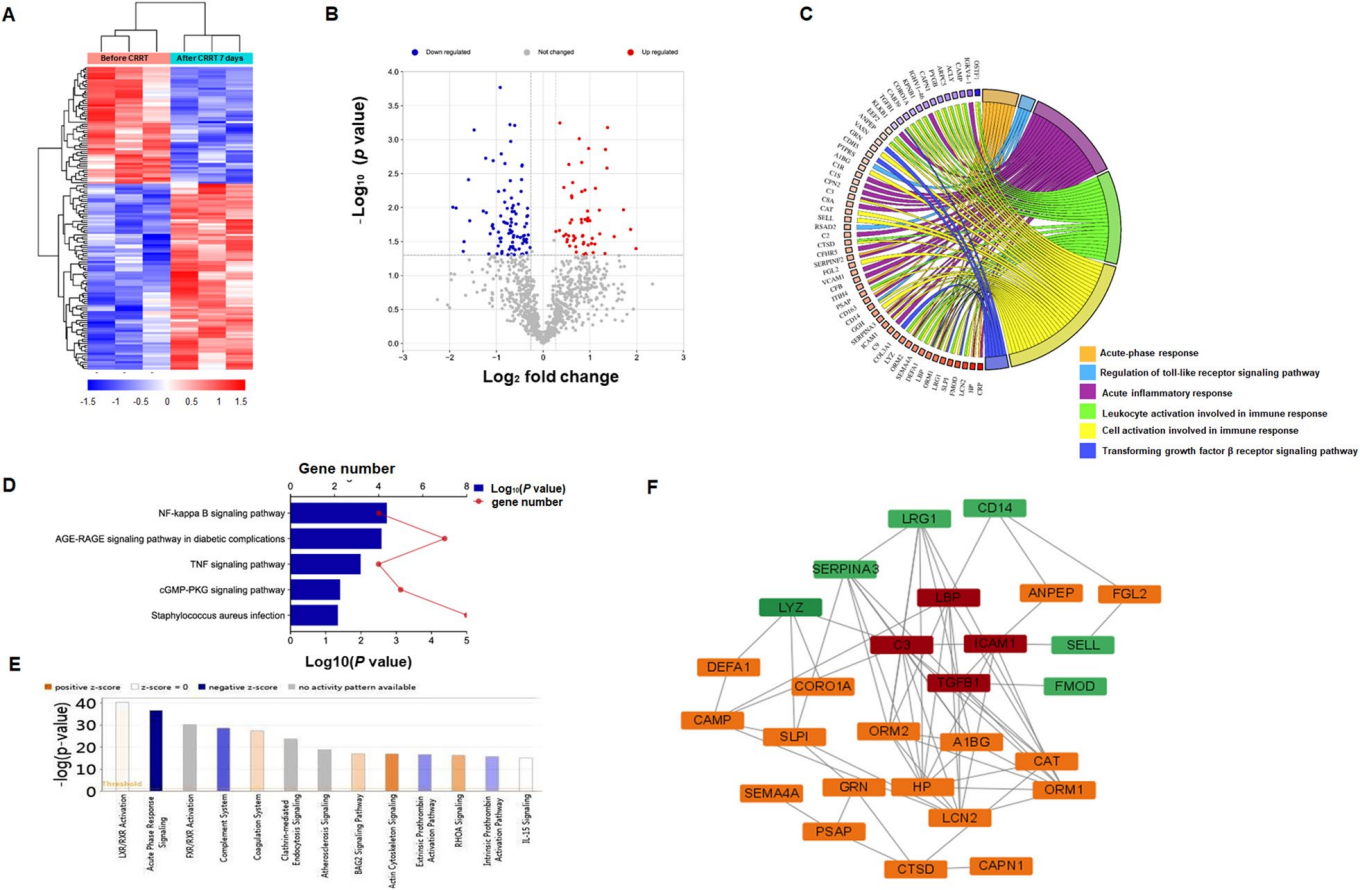
Serum proteomics analysis in 3 pediatric patients with severesepsis as a discovery set. **A** Heatmap visualization of 145 differentially expressed proteins. **B** Volcano plot of differentially expressed proteins. **C** Distribution of altered proteins for biological processes. Only GO terms that were significantly overrepresented (*p* < 0.05) are shown. **D** Significantly enriched pathways identified by KEGG pathway analysis (*p* < 0.05). **E** The canonical pathways obtained by IPA analysis. The Y-axis represents the ratio of the number of genes from the dataset that map to the pathway and the number of all known genes ascribed to the pathway. **F** Interconnections between differentially expressed proteins in the process of cell activation involved in immune response

There were 145 differentially expressed proteins identified comparing before and after CRRT for 7 days. Among them, 56 proteins were increased and 89 were decreased expression. Heatmap visualization and a volcano plot of 145 differentially expressed proteins was displayed (Fig. 2A, B). Then, Gene Ontology (GO) analysis revealed that the differentially expressed proteins participated in various biological progress including acute-phase response, acute inflammatory response, leukocyte activation involved in immune response, cell activation involved in immune response, transforming growth factor β(TGFβ) receptor signaling pathway (Fig. 2C, Table 2). Moreover, KEGG pathway analysis indicated that NF-κB signaling pathway, AGE-RAGE signaling pathway, cGMP-PKG signaling pathway, *staphylococcus aureus* infection, TNF signaling pathway were involved in the process of CRRT-mediated sepsis recovery (Fig. 2D). Furthermore, IPA for the 145 differentially expressed proteins showed that the top 5significantly different canonical pathways were LXR/RXR and FXR/RXR activation, acute phase response signaling, complement system, coagulation system (Fig. 2E). Given the important roles of immune response in sepsis, the differentially expressed protein enriched in cell activation involved in immune response were further analyzed via STRING database. Finally, interactome analysis indicated LYZ, LRG1, FMOD, SERPINA3, SELL, and CD14 could be novel candidates involved in the regulation of complement system (shown as C3 in Fig. 2F), acute-phase response (shown as LPS binding protein [LBP] in Fig. 2F), and TGFβ receptor signaling pathway (shown as TGFβ1 in Fig. 2F).

**Table 2:** The biological processes enriched by differential expressed proteins

GO_ID	Term	Differential expressed proteins	P value
GO:0006953	Acute-phase response	ITIH4, SERPINA3, HP, LBP, SERPINF2, CRP, CD163, ORM1, ORM2	< 0.001
GO:0034121	Regulation of toll-like receptor signaling pathway	CD14, LBP, RSAD2, PTPRS	0.005
GO:0002526	Acute inflammatory response	C3, ICAM1, KLKB1, ITIH4, SERPINA3, HP, LBP, SERPINF2, CRP, CD163, ORM1, ORM2, CFB, C1R, C2, C9, C1S, C8A, CPN2, CFHR5, IGKV4-1, IGHV1-46, VCAM1	0.005
GO:0002366	Leukocyte activation involved in immune response	LBP, ORM1, LCN2, CTSD, PYGB, KPNB1, LYZ, ORM2, ARPC5, CAPN1, CAB39, EEF2, DEFA1, PSAP, CAMP, OSTF1, ACLY, ANPEP, SLPI, CORO1A, SEMA4A, TGFB1	0.007
GO:0002263	Cell activation involved in immune response	GRN, LBP, ICAM1, FGL2, C3, SERPINA3, HP, A1BG, CAT, LRG1, CD14, GGH, ORM1, LCN2, CTSD, PYGB, KPNB1, LYZ, ORM2, ARPC5, CAPN1, CAB39, EEF2, DEFA1, PSAP, CAMP, OSTF1, ACLY, ANPEP, SLPI, CORO1A, SEMA4A, TGFB1, FMOD, SELL	0.009
GO:0007179	Transforming growth factor β (TGFβ) receptor signaling pathway	FMOD, TGFB1, LRG1, VASN, COL3A1, CDH5	0.044

### Confirmed analysis of serum candidates including LYZ, LRG1, FMOD, SERPINA3, SELL, andCD14 in the validation set

**Fig. 3:**
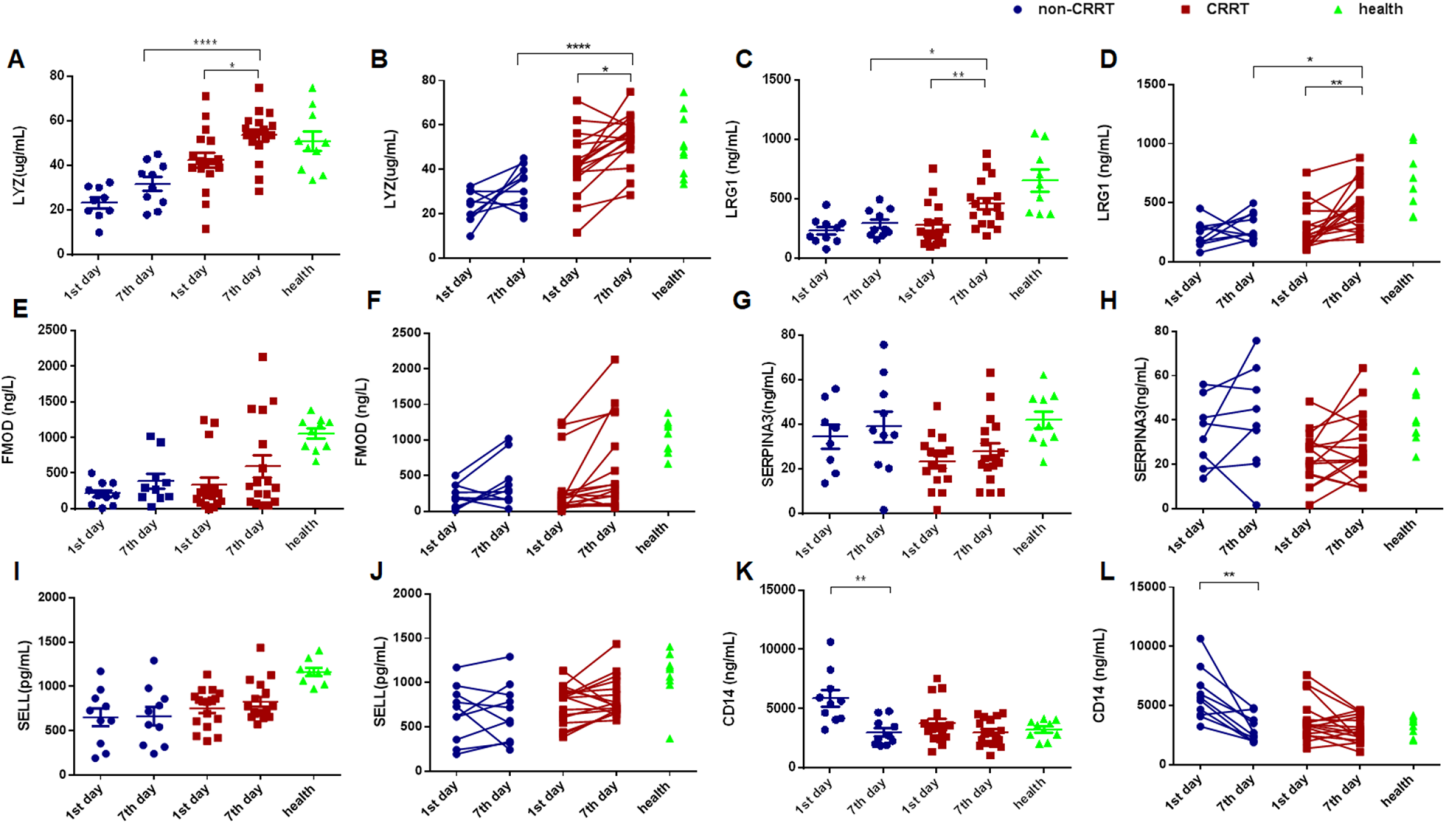
Confirmed analysis of serum candidates including LYZ, LRG1, FMOD, SERPINA3, SELL, and CD14 using ELISA in the validation set. **A**, **B** serum LYZ levels, **C**, **D** serum LRG1 levels, **E**, **F** serum FMOD levels, **G**, **H** serum SEPRINA3 levels, **I**, **J** serum SELL levels, **K**, **L** serum CD14 levels. ^*^ indicates *P* < 0.05. ^**^ indicates *P* < 0.01. ^***^indicates *P* < 0.001

As shown in Fig. 3A, B, serum level of LYZ significantly increased on the 7th day compared with the 1stday in the CRRT group (*p* < 0.05) but not CRRT group (*p* > 0.05), and serum LYZ levels were significantly higher on the 7th day in the CRRT group than the non-CRRT group (*p* < 0.001). In addition, serum levels of LRG1 (Fig. 3C, D) significantly increased on the 7th day compared with the 1st day in the CRRT group (*p* < 0.01) but not CRRT group (*p* > 0.05), and serum LRG1 levels were significantly higher on the 7th day in the CRRT group than the non-CRRT group (*p* < 0.05).However, serum levels of FMOD, SERPINA3, and SELL were no significant changes on the 7th day compared with the 1st day either in the CRRT group or the non-CRRT group (Fig. 3E–J). Importantly, serum CD14 levels decreased on the 7th day compared with the 1st day in the non-CRRT group, but not in the CRRT group (Fig. 3K, L).

### Comparison of the levels of serum novel biomarkers in CRRT responders and non-CRRT responders

**Fig. 4:**
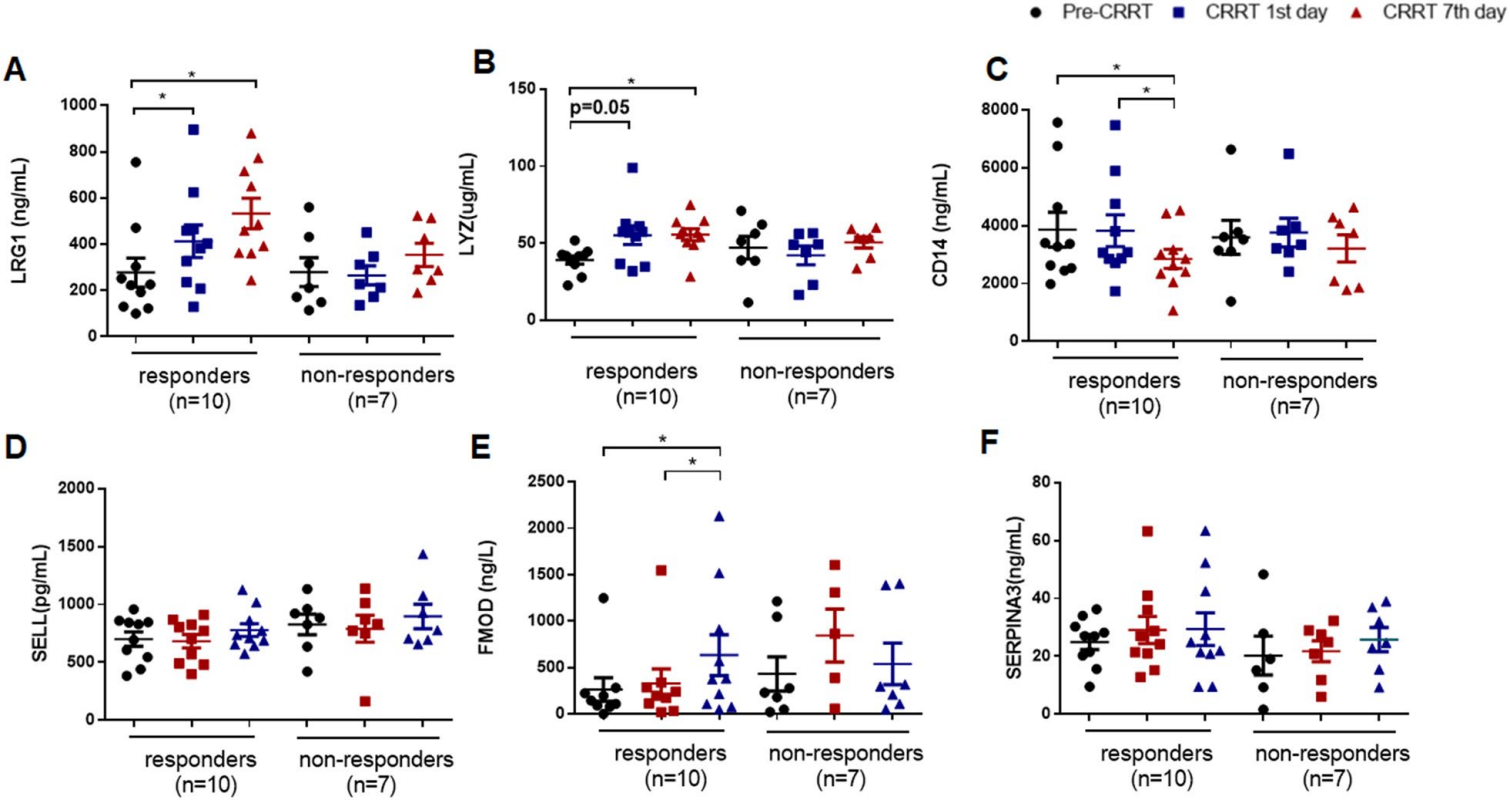
The levels of serum novel biomarkers in both CRRT responders (n = 10) and non-responders (n = 7). **A** LRG1, **B** LYZ, **(C)** CD14, **D** SELL, **E** FMOD, **F** SERPINA3. ^*^ indicates *P* < 0.05

Serum LRG1 levels were appeared significantly elevated on the CRRT 1st day compared with CRRT initiation (413.257 ng/mL *vs.* 277.824 ng/mL, P = 0.047), and serum LYZ levels changed with the similar tendency (55.233 μg/mL *vs.* 39.05 μg/mL, P = 0.05) (Fig. 4A, B). However, there were no significantly changes in serum levels of CD14, SELL, FMOD, and SERPINA3 (Fig. 4C–F). On CRRT 7th day, serum LRG1, LYZ, and FMOD levels were significantly increased compared with pre-CRRT (Fig. 4A, B, E), but serum CD14 levels were significantly decreased (Fig. 4C). However, there were no obvious changes during CRRT in non-responders (Fig. 4).

### Correlation analysis of serum biomarkers involved in sepsis and CRRT treatment to clinical indexes in pediatric patients with sepsis

**Fig. 5:**
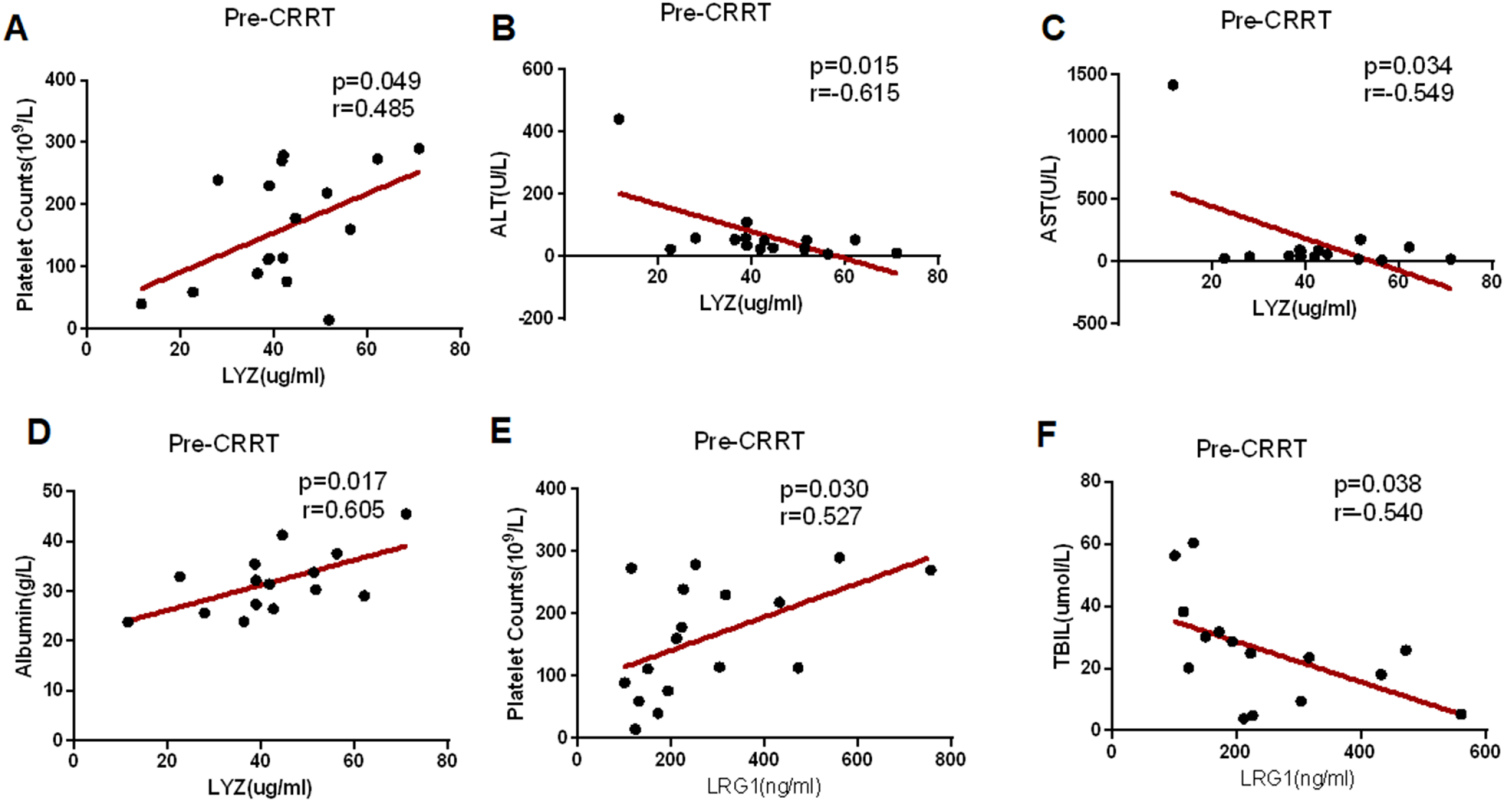
Correlation analysis of serum biomarkers and clinical indexes in pediatric patients with sepsis in the CRRT group. **A**–**D** Correlation analysis between serum LYZ level and platelet count, AST, ALT, or ALB in the CRRT group. **E**–**F** Correlation analysis between serum LRG1 level and total platelet count or TBIL

To further explore the potential roles of confirmed serum LYZ and LRG1 in sepsis and for assessing the clinical benefits of CRRT, Pearson’s correlation analysis was performed between either LYZ or LRG1 and the clinical indexes on the 1stday.In the CRRT group, serum LYZ level was positively correlated to platelet count (Fig. 5A) and level of albumin (Fig. 5D), but negatively correlated to ALT and AST (Fig. 5B, C). Similarly, serum LRG level was positively correlated to total platelet count (Fig. 5E) and negatively correlated to total bilirubin level (Fig. 5F). However, there was no correlation between these two candidates and clinical parameters in the non-CRRT group (Table 3).

**Table 3:** The correlation analysis between candidate indicators and clinical parameters in the CRRT group and the non-CRRT group

Parameters	LYZ				LRG1			
	non-CRRT		CRRT		non-CRRT		CRRT	
	*r*	*p*	*r*	*p*	*r*	*p*	*r*	*p*
PRISM III	0.582	0.225	− 0.552	0.063	0.753	0.084	− 0.423	0.171
PLT	0.393	0.261	0.485	0.049^a^	0.112	0.759	0.527	0.03^a^
WBC	− 0.055	0.880	0.293	0.270	− 0.164	0.651	0.150	0.580
TBIL	− 0.188	0.603	− 0.422	0.118	− 0.383	0.274	− 0.540	0.038^a^
ALT	− 0.057	0.875	− 0.615	0.015^a^	0.352	0.319	− 0.215	0.442
AST	− 0.057	0.884	− 0.549	0.034^a^	0.300	0.432	− 0.211	0.450
Cr	0.466	0.175	0.023	0.935	0.174	0.632	0.000	0.999
BUN	0.232	0.519	0.028	0.921	0.060	0.869	− 0.016	0.954
Alb	− 0.245	0.496	0.605	0.017^a^	0.194	0.592	0.501	0.057
CRP	− 0.038	0.917	− 0.104	0.691	− 0.344	0.330	− 0.354	0.163
PCT	− 0.190	0.598	− 0.090	0.732	− 0.123	0.736	− 0.332	0.193

*PRISM III* pediatric risk of mortality III, *PLT* platelet, *WBC* white blood cell, *TBIL* bilirubin, *ALT* alanine aminotransferase, *AST* aspartate aminotransferase, *Cr* creatinine, *BUN* blood urea nitrogen, *Alb* albumin, *CRP* c-reactive protein, *PCT* procalcitonin

^a^*P* < 0.05

### PPI network analysis of confirmed biomarkers of both LYZ and LRG1

**Fig. 6:**
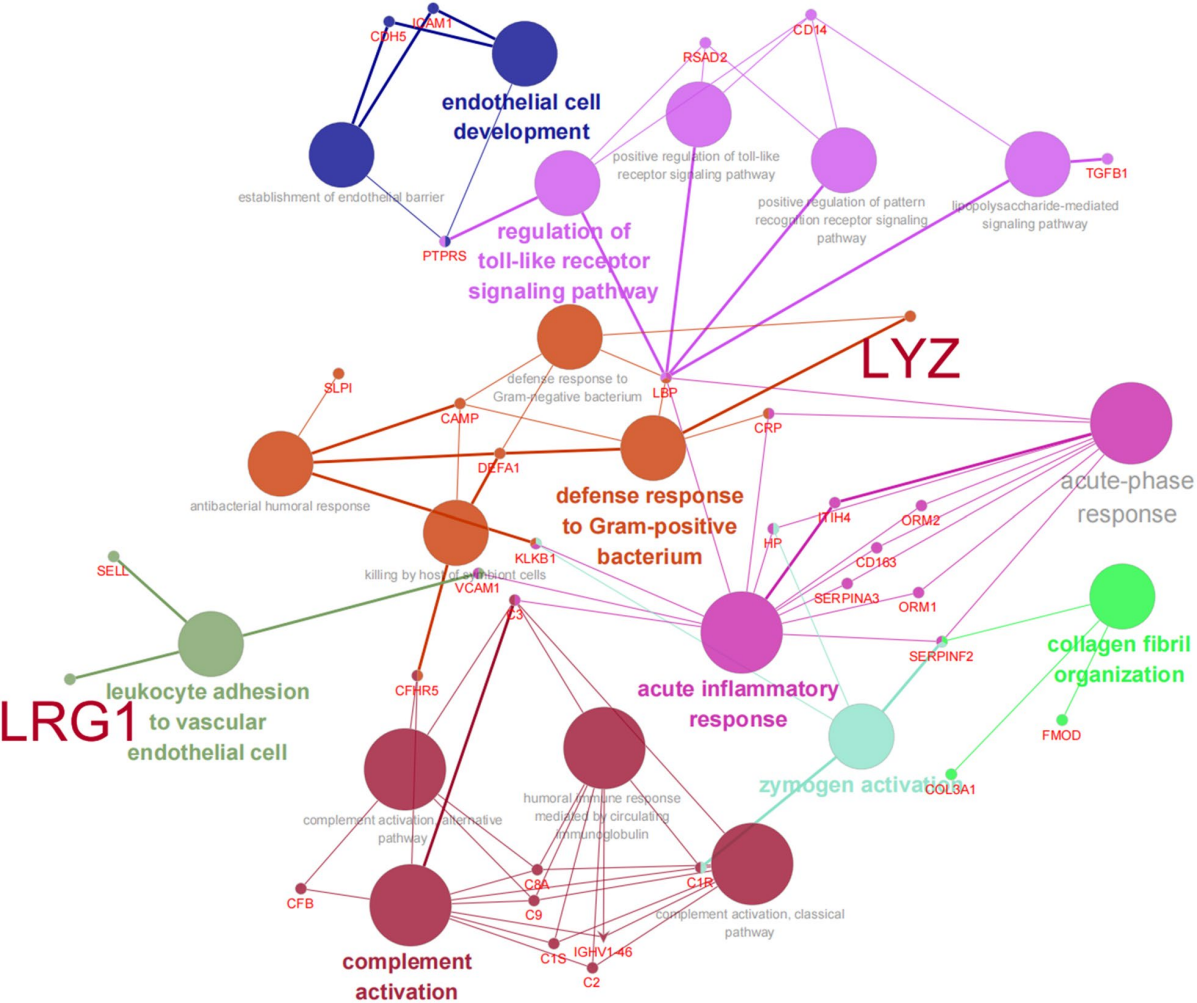
The protein–protein analysis using STRING software online

Focusing on the potential interaction network of LYZ and LRG1, leucocytes adhesion to vascular endothelial cell, acute inflammatory response, defense response to G^+^ bacterium, regulation of Toll-like receptor signaling pathway, as well as complement activation were the most important, which influenced each other; the remaining other biomarkers including CD14, FMOD, SERPINA3, and SELL had also involved into these process with potential interaction with TGFβ, CRP, LBP, etc. (Fig. 6).

## Discussion

The increasing application of CRRT in critically ill has raised a major issue about the novel biomarker to assess the adjuvant therapeutical benefits. We performed the comparison analysis of serum proteomics profile before CRRT and after CRRT for 7 days in pediatric patients with sepsis. The screened candidates were further confirmed in a larger-scale population including health control, non-CRRT group, and CRRT group. This pilot study indicated that serum LYZ and LRG1 levels were significantly associated with sepsis recovery contributed by CRRT in pediatric patients.

Serum or plasma is valuable as a reservoir of secreted proteins reflecting the patient’s pathological status and contains biomarkers of disease and treatment response [21, 22]. Comprehensive determination of the serum proteome of septic patients may help better understand the pathogenesis of sepsis as well as identify the novel biomarkers for assessing the outcome or therapeutic effect. CRRT is suggested as an adjuvant therapy for pediatric sepsis by 2020 SSC guideline for children [9]. However, the comprehensive impact of CRRT in pediatric sepsis is largely unknown until now. To the best our knowledge, it is the first report about the changes of serum proteomic profile and related integrated signaling pathway involved in CRRT-mediated adjuvant therapy in pediatric sepsis.

Gong Y et al. [23] reported that a proteomic approach identified 34 differentially expressed proteins before CRRT (0 h) and at 24 h, 48 h and 72 h during CRRT in 20 adults with sepsis. In our present study, TMT-labeled peptides were analyzed by LC–MS/MS, and 145 differentially expressed proteins were identified. Furthermore, the integrated signaling pathway based on the differentially expressed proteins was obtained using GO analysis, IPA and protein–protein interaction analysis on STRING database. All significantly involved signaling pathways included acute-phase response, regulation of toll-like receptor (TLR) signaling pathway, acute inflammatory response, leukocyte activation and cell activation involved in immune response, and TGFβ receptor signaling pathway. Moreover, complements system was another integrated pathway identified by IPA. It is well known that activation of complements, for example, complement component 3 (C3) and C5, is involved in the development of septic shock, and the loss or inhibition of complement components affects the immune process including phagocytosis, autophagy and inflammatory immune response [24, 25]. Lipopolysaccharide (LPS)-binding protein (LBP) functions as an enhancer of the host response to LPS. The LPS-LBP complex then binds to a receptor complex including the CD14 and toll-like receptor 4 (TLR4) proteins, which triggers the secretion of pro-inflammatory cytokine [26, 27]. Given the crucial roles of C3 and LBP, we integrated the differentially expressed proteins and focused on the potential regulators of C3 and LBP.

In another aspect, TGFβ1 concentrations in serum and BALF were significantly decreased in patients with early initiation of CRRT compared to those with late initiation of CRRT on day 2 and day 7, and early initiation of CRRT is associated with favorable clinical outcomes in ARDS patients, which might be due to the reduced serum and BALF TGFβ1levels through CRRT [28]. So, we also paid close attention to the potential regulators of TGFβ. Finally, LYZ, LRG1, SERPINA3, SELL, and CD14 were selected as potential regulators of C3 or LBP, and FMOD was chosen as only potential regulator of TGFβ. Moreover, the PPI interaction network suggests that all these novel biomarkers associated with CRRT would be involved in the process of host-response with inflammatory response, leucocytes adhesion to vascular endothelial cell, as well as complement activation. Though confirmed in a larger-scale population, LYZ and LRG1 was screened as novel biomarkers of sepsis recovery contributed by CRRT as an adjuvant therapy. To date, only a limited number of serum proteins have been studied in adults received CRRT [23, 28]. Here, we firstly report about the potential novel biomarker involved in both sepsis recovery and CRRT response in pediatric sepsis.

No matter in the CRRT group or the non-CRRT group, Serum LYZ levels were increased on the 7th day compared with the1st day, which suggests that elevated serum LYZ levels may be associated with sepsis recovery. Moreover, serum LYZ levels were higher in the CRRT group compared with non-CRRT group. So, the clinical benefits of CRRT might related to altering serum LYZ levels. Consistently, Zhang et al. [29] also identified LYZ as a potential biomarker for sepsis by analyzing gene expression profiles. Moreover, correlation analysis also showed that serum LYZ levels in patients were correlated with the total number of platelets, AST, ALT, and Alb. This suggests that LYZ may affect the pathogenesis of sepsis by affecting coagulation, liver function, or fluid leakage. Considering the novelty of LYZ as an indicator of sepsis recovery and clinical benefits of CRRT, the underlying mechanisms of LYZ involved in roles of CRRT in pediatric sepsis is worth of more attention in the future. In addition, several studies have shown that SELL [15] and SERPINA3 [30] are also associated with sepsis, but it was not confirmed in our present study.

As same as serum LYZ levels, serum LRG1 levels were significantly higher on the 7th day after CRRT than before CRRT, and serum LRG1 levels in patients who received CRRT were significantly higher than that of the non-CRRT group. Moreover, serum LYZ and LRG1 were significantly changed in CRRT responders, but not CRRT non-responders. Furthermore, the influence of CRRT application in the levels of serum LYZ, LRG1, CD14, FMOD displays more long-term effects until 7th day after CRRT initiation. Our recent study indicated that CRRT plays a key role in regulation of polymorphonuclear myeloid-derived suppressor cell expansion [31]. All these results suggested that besides of the direct effects of the filter under CRRT support, the indirect effects of CRRT on sepsis progression via host-response might be worth more attention in the future. So, we can suspect that serum LRG1 level could be another biomarker which is responsible for the clinical benefits of CRRT in pediatric sepsis. There were evidences indicating that LRG1 regulates endothelial TGF-β signaling [32] and CRRT can reduce serum level of TGF-b [26]. All these results prompted the possibility of the association between LRG1 levels and CRRT. However, Tomoaki et al. [33] reported that serum LRG1 level in patients with sepsis (n = 10) was higher than that of non-sepsis patients (n = 10) (*p* < 0.05). The different changes during sepsis recovery between our study and Tomoaki’s report might due to the difference of subjects’ selection, severity of sepsis, mode of CRRT, or different methods for detection.

CD14, a well-known myeloid cell marker, a glycosylphosphatidylinositol (GPI)-anchored membrane protein, is critical for ensuring optimal TLR4 response to LPS and macrophage production of pro-inflammatory cytokines [34]. Previous study reported that elevated levels of soluble CD14 in different fluids can be detected during sepsis [35, 36], The findings of this study are consistent with previous reports that elevated levels of soluble CD14 in different fluids can be detected during sepsis. In the validation cohort, non-CRRT group, the level of serum CD14 in sepsis patients on the 7th day after treatment was significantly lower than that in 24 h after admission in PICU. There was also a slight decrease in CD14 levels in the CRRT group. These results suggests that elevated serum CD14 levels might be involved in the development of sepsis, and decreased serum CD14 level could be an indicator of sepsis recovery. In the present study, there was no sufficient evidence to prove the clinical benefits of CRRT in aspect of decreasing serum CD14 levels.

There are several limitations. Firstly, this study was a pilot study for proteomics analysis, and the sample size was small. So, a prospective observational study with a larger population is required to strengthen the conclusion of this study. Secondly, among screened 6 proteins, only LYZ and LRG1 were validated to be potential biomarker for assessing the progression of sepsis and clinical benefits of CRRT in pediatric patients with sepsis. There is possible for further exploration about the novel biomarkers. Thirdly, the general significance of these novel biomarkers would be confirmed in a larger population with sepsis. Fourthly, we did not collect the blood sample each day after CRRT initiation due to the limited blood sample for children. All these issues need further studies in the future.

## Conclusion

Based on the serum proteomic analysis in patients with sepsis before and after CRRT, elevated serum LYZ and LRG1 levels are associated with clinical benefits of CRRT during sepsis recovery. LYZ and LRG1 might be valuable indicators for assessing the clinical benefits of CRRT in pediatric patients with severe sepsis.

## Supplementary Information

**Additional file 1: Table S1.** The values of pSOFA score in patients with severe sepsis on pre-CRRT, 1st day, and 7th day after CRRT initiation. **Table S2.** The values of pSOFA in patients with severe sepsis on pre-CRRT, 1st day, and 7th day after CRRT initiation.

## Data Availability

All data generated or analyzed during this study are included in the manuscript and the additional files. Any further information is available from the corresponding author on request.

## Abbreviations

CRRT: Continuous renal replacement therapy
LYZ: Lysozyme C
LRG: Leucine-rich alpha-2-glycoprotein;
FMOD: Fibromodulin
SERPINA3: Alpha-1-antichymotrypsin
SELL: L-selectin
CD14: Monocyte differentiation antigen CD14
PRISMIII: Pediatric risk of mortality III
CRP: C-reactive protein
WBC: White blood cells
PCT: Procalcitonin
ALT: Alanine aminotransferase
TBIL: Bilirubin
γ-GT: γ-glutamyl transpeptidase
Fib: Fibrinogen
APTT: Activated partial thromboplastin time
INR: International normalized ratio
PT: Prothrombin time
PLT: Platelet
Cr: Creatinine
BUN: Blood urea nitrogen
Alb: Albumin
Lac: Lactate
