# Supplementary material for: The novel biomarkers for assessing clinical benefits of continuous renal replacement therapy in pediatric sepsis: a pilot study
Source: Clin Proteomics. 2023 Jan 18;20:4. doi: 10.1186/s12014-023-09392-2 (PMC9847018; doi:10.1186/s12014-023-09392-2)
Supplement: Supplementary file 1 — Additional file 1: Table S1. The values of pSOFA score in patients with severe sepsis on pre-CRRT, 1st day, and 7th day after CRRT initiation. Table S2. The values of pSOFA in patients with severe sepsis on pre-CRRT, 1st day, and 7th day after CRRT initiation. [file 12014_2023_9392_MOESM1_ESM.docx]

**Additional file 1: Table S1. The values of pSOFA score in patients with severe sepsis on pre-CRRT, 1^st^ day, and 7^th^ day after CRRT initiation**

|  | pSOFA score | | | | | | |
| --- | --- | --- | --- | --- | --- | --- | --- |
|  | CRRT group | | |  | Non-CRRT group | | |
| Case | pre-CRRT | CRRT 1^st^ day | CRRT 7^th^ day | Case | on admission | 1^st^ day | 7^th^ day |
| No.1 | 8 | 6 | 6 | No.1 | 3 | 4 | 0 |
| No.2 | 12 | 7 | 2 | No.2 | 5 | 6 | 2 |
| No.3 | 5 | 7 | 3 | No.3 | 7 | 8 | 0 |
| No.4 | 9 | 7 | 7 | No.4 | 10 | 9 | 3 |
| No.5 | 3 | 4 | 0 | No.5 | 12 | 12 | 9 |
| No.6 | 11 | 6 | 4 | No.6 | 4 | 4 | 2 |
| No.7 | 9 | 7 | 5 | No.7 | 9 | 8 | 3 |
| No.8 | 6 | 9 | 6 | No.8 | 7 | 6 | 1 |
| No.9 | 13 | 8 | 6 | No.9 | 9 | 7 | 4 |
| No.10 | 6 | 6 | 7 | No.10 | 7 | 7 | 3 |
| No.11 | 8 | 6 | 7 |  |  |  |  |
| No.12 | 9 | 11 | 2 |  |  |  |  |
| No.13 | 8 | 6 | 3 |  |  |  |  |
| No.14 | 6 | 4 | 0 |  |  |  |  |
| No.15 | 2 | 3 | 0 |  |  |  |  |
| No.16 | 12 | 9 | 0 |  |  |  |  |
| No.17 | 5 | 6 | 1 |  |  |  |  |

**Additional file 1: Table S2. The values of pSOFA in patients with severe sepsis on pre-CRRT, 1^st^ day, and 7^th^ day after CRRT initiation**

| n | pSOFA | | | Responder |  |
| --- | --- | --- | --- | --- | --- |
|  | pre-CRRT | CRRT 1^st^ day | CRRT 7^th^ day | Yes | No |
| 1 | 8 | 6 | 6 | √ |  |
| 2 | 12 | 7 | 2 | √ |  |
| 3 | 5 | 7 | 3 |  | √ |
| 4 | 9 | 7 | 7 | √ |  |
| 5 | 3 | 4 | 0 |  | √ |
| 6 | 11 | 6 | 4 | √ |  |
| 7 | 9 | 7 | 5 | √ |  |
| 8 | 6 | 9 | 6 |  | √ |
| 9 | 13 | 8 | 6 | √ |  |
| 10 | 6 | 6 | 7 |  | √ |
| 11 | 8 | 6 | 7 | √ |  |
| 12 | 9 | 11 | 2 |  | √ |
| 13 | 8 | 6 | 3 | √ |  |
| 14 | 6 | 4 | 0 | √ |  |
| 15 | 2 | 3 | 0 |  | √ |
| 16 | 12 | 9 | 0 | √ |  |
| 17 | 5 | 6 | 1 |  | √ |
